# Topic Modeling of Nursing Documentation in Hemodialysis Units: A Mixed‐Methods Study of Nursing Surveillance Activities

**DOI:** 10.1155/jonm/5445706

**Published:** 2026-05-25

**Authors:** Mi-Kyoung Cho, Hye-Young Kim, Hyohjung Lee, Yoon Hee Cho

**Affiliations:** ^1^ Department of Nursing Science, Research Institute of Nursing Science, College of Nursing, Chungbuk National University, Cheongju, South Korea, chungbuk.ac.kr; ^2^ Department of Internal Medicine, Chungbuk National University Hospital, College of Medicine, Chungbuk National University, Cheongju, South Korea, chungbuk.ac.kr; ^3^ Department of Nursing Science, Research Institute of Nursing Science, College of Nursing, Chungbuk National University, Cheongju, South Korea, chungbuk.ac.kr; ^4^ Department of Nursing, College of Nursing, Dankook University, Cheonan, South Korea, dankook.ac.kr

**Keywords:** machine, natural language processing, nursing records, patient safety, pattern analysis, renal dialysis

## Abstract

**Aim and Objectives:**

This study aimed to characterize documentation patterns in hemodialysis nursing records using integrated text mining and nursing surveillance analysis, focusing on surveillance priorities and documentation imbalances.

**Background:**

Hemodialysis patients require continuous nursing surveillance due to frequent intradialytic complications; however, systematic analyses of nursing documentation in this setting remain limited.

**Design:**

A mixed‐methods study integrating quantitative text mining and structured analysis of nursing surveillance activities.

**Methods:**

Free‐text nursing records (300,321 entries) generated during maintenance hemodialysis sessions for 188 adult patients at a tertiary hospital in South Korea (July 2020–June 2025) were analyzed. Keyword frequency analysis and latent Dirichlet allocation topic modeling were performed after preprocessing, resulting in an eight‐topic model. Nursing surveillance activities were mapped to the 16‐item Korean Nursing Surveillance Scale through sentence‐level classification by two independent researchers.

**Results:**

The top 30 keywords accounted for 75.3% of all extracted terms, indicating highly repetitive documentation. Terms related to dialysis procedures, vascular access, blood pressure, vital signs, bleeding, falls, pain, dizziness, and hypotension were frequently documented, whereas education‐related terms were less frequently documented. Topic modeling identified three overarching domains: the hemodialysis treatment process, clinical assessment and monitoring, and safety and risk management. Surveillance item analysis demonstrated high documentation frequencies for vascular access, vital signs, bleeding, and hypotension or dizziness, with low documentation of patient self‐management and education.

**Conclusion:**

Hemodialysis nursing documentation primarily emphasizes physiological surveillance and safety management, with relatively limited documentation of patient education and self‐management support.

**Implications for Nurse Leaders:**

These findings suggest that optimizing electronic nursing record templates, along with incorporating structured prompts and natural language processing approaches, may support more balanced documentation and data‐driven decision‐making.

## 1. Introduction

The global burden of chronic kidney disease (CKD) has increased significantly in recent decades. According to the Global Burden of Disease Study, the number of people living with CKD nearly doubled between 1990 and 2017, reaching approximately 700 million worldwide [1]. As the prevalence of CKD continues to rise worldwide, the number of patients progressing to end‐stage renal disease (ESRD) and requiring maintenance hemodialysis is also increasing.

Hemodialysis aims to remove waste products and maintain physiological stability; however, a wide range of intradialytic complications frequently occur during the treatment process [2]. During dialysis, symptoms such as hypotension, muscle cramps, nausea, headache, and chest pain hinder patient cooperation and compromise treatment compliance [3–5]. These symptoms can lead to serious consequences if not promptly recognized and managed [3, 5]. Close observation and interpretation of the patient condition are essential [6]. In this context, nursing surveillance (NS) is a core activity that involves systematic observation and interpretation of changes in patient condition. It enables early recognition of potential risks and plays a key role in ensuring patient safety in hemodialysis care.

Hemodialysis‐related nursing records are generated throughout the treatment process [7]. These records provide real‐time documentation of dialysis care, including dialyzer settings, vital signs, weight changes, patient responses, adverse events, and nursing interventions [8]. They also reflect nurses’ clinical judgment and decision‐making processes [6, 8].

The primary goal of nursing documentation is to support patient‐centered care by systematically recording patient status, nursing interventions, and outcomes [9, 10]. By documenting nurses’ judgments and interventions [9], these records support rapid identification of changes in patient status and improve continuity and quality of care [11]. In particular, documentation within electronic health records (EHRs) facilitates efficient management of nursing records. Furthermore, it supports data‐driven analysis to improve nursing outcomes based on systematically recorded patient clinical characteristics [9, 11, 12]. Therefore, analyzing these records can be a useful strategy for identifying common patient problems and solutions, nurse interventions, and patient responses. Previous studies have shown that such documentation supports nurses’ critical thinking, clinical judgment, and professional development [13].

One prior study analyzed free‐form nursing records of hospitalized patients to identify topics such as “patient well‐being,” “patient transport and nursing activities,” and “treatment and pain management,” providing insights into nurses’ activities for hospitalized patients [14]. Another study analyzed emergency room nursing records to highlight the importance of continuous monitoring and surveillance activities, suggesting that NS plays a key role in ensuring patient safety [11]. However, despite these contributions, previous studies have primarily focused on general inpatient or emergency care settings and have largely examined the descriptive content of nursing documentation rather than explicitly conceptualizing and analyzing NS as a distinct clinical function [15]. Moreover, systematic investigations of NS in high‐risk, specialized settings—such as hemodialysis units, where rapid physiological changes frequently occur—remain limited [16]. In addition, although nursing records are inherently unstructured and complex, few studies have applied advanced text‐mining approaches, such as latent Dirichlet allocation (LDA), to identify the latent thematic structure underlying nursing documentation [14, 17]. Therefore, a clear gap exists in understanding how NS is manifested within hemodialysis nursing records and how such patterns can be systematically identified using data‐driven analytical methods.

Beyond its clinical significance, nursing documentation in high‐risk settings such as hemodialysis is closely linked to key challenges faced by nurse leaders, including ensuring patient safety, maintaining documentation quality, and standardizing care processes across complex clinical environments. Despite its importance, limited research has examined how patterns in nursing documentation can inform leadership decision‐making and quality management. By analyzing documentation patterns and imbalances using data‐driven approaches, this study provides actionable insights into how clinical priorities and system‐level factors are reflected in nursing records. Therefore, a clearer understanding of these patterns may support nurse managers in monitoring care quality, identifying gaps in documentation practices, and optimizing care delivery and organizational performance.

The LDA topic model is a type of unsupervised learning model that facilitates summarization, exploration, and retrieval of large, unstructured document collections, such as nursing records [18]. Therefore, in this study, we applied LDA topic modeling to nursing records from a hemodialysis ward to analyze the symptoms, nursing activities, and responses of hemodialysis patients. We also aimed to elucidate the role of nurses through an in‐depth analysis of their nursing monitoring activities. By integrating the concept of NS with advanced text‐mining techniques, this study seeks to provide a novel perspective on how nurses detect, interpret, and respond to patient conditions in hemodialysis settings.

This study aims to (1) identify the most frequently documented keywords in hemodialysis nursing records, (2) explore the latent thematic structure of nursing notes using LDA topic modeling, and (3) quantify the frequency and types of NS activities reflected in these records.

## 2. Methods

### 2.1. Study Design

This study employed a mixed‐methods design integrating quantitative text mining and structured analysis of documentary data. Natural language processing–based topic modeling using LDA was applied to free‐text nursing records to identify latent thematic structures. The identified topics were then interpreted and categorized based on NS domains, and each topic was assigned a label through an iterative review process. In addition, documented NS activities were systematically classified using a structured framework to examine their types and frequencies in the hemodialysis setting. This study was reported in accordance with the Strengthening the Reporting of Observational Studies in Epidemiology (STROBE) checklist for cross‐sectional studies (Supporting Information 1).

### 2.2. Study Participants and Data

The study participants were adult patients receiving maintenance hemodialysis in the hemodialysis unit of a tertiary general hospital in South Korea. Rather than directly recruiting individual patients, this study analyzed nursing records generated during the hemodialysis process. All nursing documentation created during hemodialysis sessions was used as the primary data source, and the unit of analysis varied depending on the analytical procedure. This study included all nursing records generated by EHR from July 2020 to June 2025. A total of 188 patients who received maintenance hemodialysis during that period were included in the study, and a total of 300,321 records were generated during the study period.

### 2.3. Data Analysis

Data were analyzed using SPSS Version 29.0 (IBM Corp., Armonk, NY, USA), Python (Google Colaboratory, Google LLC, Mountain View, CA, USA), KoNLPy (an open‐source Korean natural language processing library), Gensim, and Microsoft Excel (Microsoft Corp., Redmond, WA, USA). Descriptive statistics, including frequencies, percentages, means, and standard deviations, were used to summarize the general characteristics of the study sample using SPSS.

Free‐text nursing records documented during hemodialysis sessions were preprocessed prior to topic modeling. Text cleaning involved the removal of special characters, redundant symbols, and extra spaces. Semantically equivalent expressions commonly used in clinical practice were standardized to a single representative term to ensure consistency across records. To enhance clinical relevance, a user‐defined dictionary was developed by the research team to retain meaningful dialysis‐related terms while excluding noninformative words. Morphological analysis and tokenization were performed using KoNLPy’s Okt analyzer [19], with a focus on noun extraction. Term frequency was then calculated to identify commonly occurring keywords within the nursing records, and the extracted keywords were reviewed to confirm contextual appropriateness in the hemodialysis setting [20].

Topic modeling was conducted using LDA, an unsupervised probabilistic modeling approach suitable for identifying latent thematic structures in unstructured text data [21, 22]. Because nursing records often contain multiple clinical issues within a single entry, LDA was considered appropriate for capturing the multifaceted nature of hemodialysis nursing documentation [21]. A document–term matrix was constructed based on term frequency, and LDA was implemented using the gensim.models.LdaModel module. To reduce redundancy, terms appearing in more than 50% of documents were excluded using the no_above = 0.5 parameter to remove overly frequent, nondiscriminative terms that may reduce topic differentiation [23]. Model performance was evaluated using topic coherence (c_v), and the optimal number of topics was determined based on model interpretability and statistical fit [23, 24]. Topic weights were calculated as the average topic probability across all documents to assess the relative prominence of each topic. Extracted topics were interpreted based on their high‐probability keywords and reviewed by the research team to ensure clinical relevance. For keyword frequency analysis and LDA topic modeling, each nursing record was treated as a single document (document‐level unit of analysis).

NS activities documented in hemodialysis nursing records were analyzed to examine their types and frequency. NS was conceptualized as activities related to the continuous observation, assessment, interpretation, and early identification of changes in patient status during hemodialysis. For the mapping of NS activities, the 16‐item Korean Nurse Surveillance Scale developed by Kim and Cho [25] was used as the analytical framework. Two researchers independently reviewed the nursing records at the statement level (i.e., sentence‐level unit of analysis) to identify documentation corresponding to surveillance‐related activities. Inter‐rater reliability for the classification of NS activities was assessed using percent agreement and Cohen’s kappa coefficient. The percent agreement was 81.25%, and the kappa coefficient was 0.798, indicating substantial agreement between the two researchers. Discrepancies were resolved through discussion until consensus was reached. Surveillance‐related keywords were then defined and used to extract relevant records, which were organized into a mapping framework. Based on this mapping, the frequency of each type of NS activity was calculated.

### 2.4. Ethical Considerations

All research procedures were approved by the Institutional Review Board of the researchers’ institution (IRB No. CBNU‐2025‐A‐0088), and the study was conducted in accordance with the Declaration of Helsinki. Prior to analysis, the dataset was reviewed and approved by the Data Review Committee of Chungbuk National University Hospital in accordance with institutional data governance policies, together with the IRB approval and research protocol. All data were fully anonymized to prevent personal identification, and access to the data and analysis files was restricted to the research team through password protection. All study data and analytic files will be securely stored for 3 years following study completion and subsequently permanently deleted. Throughout the study, the researchers adhered to strict principles of data protection and confidentiality.

## 3. Results

### 3.1. Participant Characteristics

Table 1 presents the demographic and clinical characteristics of the participants (*n* = 188). All participants were diagnosed with end‐stage kidney disease (ESKD) on dialysis. Of the participants, 111 (59.0%) were men and 77 (41.0%) were women. The mean age was 66.7 years (SD = 11.9), with the largest proportion of participants aged 60–69 years (30.8%), followed by those aged 70–79 years (27.7%). Regarding the causes of ESKD, diabetes mellitus was the most common cause (46.3%), followed by hypertension (32.4%) and CKD of other etiologies (12.8%). Other causes included autosomal dominant polycystic kidney disease (4.3%), drug‐related nephrotoxicity (2.1%), glomerulonephritis (2.1%), and acute kidney disease (1.6%). Twenty‐one participants (11.2%) had an unknown or unrecorded cause of ESKD. Nearly all participants received hemodialysis three times per week (96.3%).

**TABLE 1:** Characteristics of the participants (*n* = 188).

Characteristics	*n*	%	*m* ± SD
Sex			
Men	111	59.0	
Women	77	41.0	
Age			
≤ 49	13	6.9	66.66 ± 11.93
50–59	37	19.7	
60–69	58	30.8	
70–79	52	27.7	
≥ 80	28	14.9	
Causes of ESKD			
Diabetes mellitus	87	46.3	
Hypertension	61	32.4	
Others (e.g. glomerulonephritis, polycystic kidney disease, drug side effects, etc.)	51	27.1	
Unknown or no record	21	11.2	
Number of dialysis sessions per week			
3 times	181	96.3	
Twice	7	3.7	

*Note:* Multiple responses are possible for the causes of ESKD.

### 3.2. High‐Frequency Keywords in Hemodialysis Nursing Documentation

Table 2 shows the top 30 most frequently occurring terms in the preprocessed hemodialysis nursing records. Key keywords included “dialysis,” “condition,” “fall,” “pain,” “blood pressure,” “education,” and “safety.” These top 30 keywords accounted for approximately 75.3% of all 1,259,314 extracted tokens. This finding suggests that hemodialysis nursing records consist of a relatively limited and repetitive set of core terms related to the treatment process, patient condition monitoring, safety, and nursing interventions.

**TABLE 2:** Top 30 frequent keywords in hemodialysis nursing records.

Rank	Keyword (Korean)	Keyword (English)	Frequency
1	투석	Dialysis	116,460
2	상태	Condition	68,319
3	낙상	Fall	59,057
4	확인	Check	42,688
5	통증	Pain	41,610
6	종료	Termination	40,075
7	시작	Initiation	39,145
8	혈관	Vascular access	38,683
9	교육	Education	38,445
10	예방	Prevention	37,769
11	증상	Symptom	34,195
12	설명	Explanation	29,191
13	침대	Bed	25,916
14	혈압	Blood pressure	23,221
15	활동	Activity	22,890
16	안내	Guidance	22,670
17	직원	Staff	22,668
18	안전	Safety	22,638
19	화재	Fire	22,635
20	대피	Evacuation	22,633
21	활력징후	Vital signs	22,568
22	수행	Performance	22,460
23	올림	Elevation	19,718
24	출혈	Bleeding	18,985
25	고정	Fixation	18,032
26	양호	Stable status	17,682
27	난간	Side rail	15,693
28	어지러움	Dizziness	14,135
29	저혈압	Hypotension	14,103
30	발생	Occurrence	14,090

### 3.3. Topic Modeling Results and Thematic Structure of Hemodialysis Nursing Records

To determine the number of topics, we repeatedly compared LDA models with different topic counts ranging from 5 to 12, referring to the topic counts and human judgment criteria presented in previous studies applying topic modeling to nursing records [14, 26]. To reduce computational burden, we used 80,000 nursing record documents randomly selected from the entire dataset. The models for each topic count were compared using a topic diversity index calculated based on the degree of overlap between the top word sets of each topic. As a result, topic diversity was similarly high at *K* = 7 and *K* = 8, and diversity tended to decrease as the number of topics increased thereafter. Among these topic models, an eight‐topic model was ultimately selected based on comprehensive consideration of quantitative indicators, topic redundancy, and clinical interpretability.

As presented in Table 3, the eight final topics were grouped into three overarching domains: symptom reporting and risk recognition (Topic 2), vascular access and clinical status assessment (Topic 3), vital signs and bleeding monitoring (Topic 8), initiation and termination of hemodialysis treatment (Topic 7), safety education and evacuation preparedness (Topic 1), activity and mobility safety management (Topic 4), bed environment configuration and positioning (Topic 5), and active nursing interventions for fall prevention (Topic 6). These themes were further grouped into three overarching domains: clinical assessment and monitoring, hemodialysis treatment process, and safety and risk management.

**TABLE 3:** Topic modeling results of hemodialysis nursing records using latent Dirichlet allocation, grouped by overarching domains.

Overarching domain	Topic (avg. weight)	Thematic label	Key keyword
Hemodialysis treatment process	Topic 7 (0.246)	Hemodialysis treatment initiation and termination	Dialysis, initiation, termination, progression, blood

Clinical assessment and monitoring	Topic 8 (0.178)	Vital signs and bleeding monitoring	Vital signs, blood pressure, bleeding, check, site
	Topic 3 (0.147)	Vascular access and clinical status assessment	Vascular access, catheter, heparin, pain, condition
	Topic 2 (0.095)	Symptom reporting and risk recognition during hemodialysis	Symptoms, dizziness, hypotension, occurrence, reporting

Safety and risk management	Topic 1 (0.101)	Safety education and evacuation preparedness	Education, guidance, fire, evacuation, preparedness
	Topic 4 (0.083)	Activity and mobility safety management	Activity, mobility, oxygen, performance
	Topic 5 (0.080)	Bed environment configuration and positioning	Bed, side rails, elevation, lowering, fixation
	Topic 6 (0.071)	Active nursing interventions for fall prevention	Falls, implementation, caution, nursing care, posting

In the clinical assessment and monitoring domain, symptom reporting and risk recognition, vascular access and clinical status assessment, and vital signs and bleeding monitoring emerged as key themes. These themes reflect nursing activities that continuously assess patients during hemodialysis and early detection of abnormalities such as dizziness, hypotension, vascular status, vital signs, and bleeding.

In the hemodialysis treatment process domain, themes related to initiation and termination of dialysis and treatment progress emerged as a single theme, representing the procedural flow of hemodialysis treatment and the involvement of healthcare professionals in the treatment process. This topic had the highest average weight among all topics, demonstrating its central role in the overall nursing record.

In the safety and risk management domain, the topics were categorized into safety education and evacuation preparedness, activity and mobility safety management, bed environment configuration and positioning, bedside environment management and posture management, and proactive nursing interventions for fall prevention. The fact that bed environment configuration and direct nursing interventions emerged as separate topics demonstrates that fall prevention in hemodialysis nursing is a multifaceted issue encompassing both environmental management and the practical actions of nurses.

**FIGURE 1:**
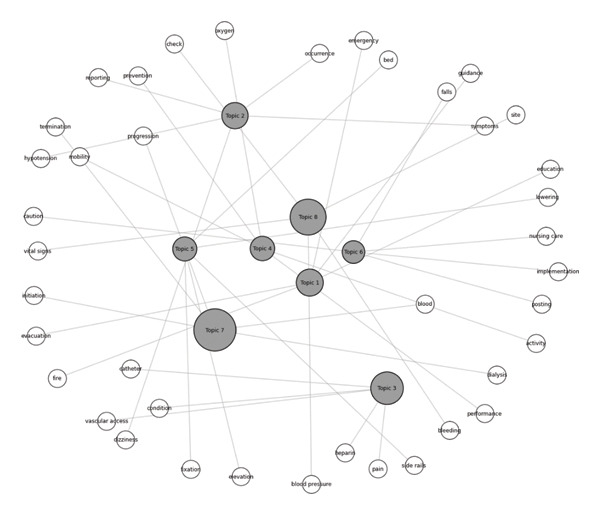
Topic–keyword network of hemodialysis nursing records. Topic 1: safety education and evacuation preparedness; Topic 2: symptom reporting and risk recognition during hemodialysis; Topic 3: vascular access and clinical status assessment; Topic 4: activity and mobility safety management; Topic 5: bed environment configuration and positioning; Topic 6: active nursing interventions for fall prevention; Topic 7: hemodialysis treatment initiation and termination; Topic 8: vital signs and bleeding monitoring.

Figure 1 illustrates the topic–keyword network derived from hemodialysis nursing records. Circular nodes represent topics and keywords, with the size of topic nodes proportional to their average weights. Keyword nodes indicate the core terms associated with each topic, and edges represent strong associations between topics and their keywords.

The topic related to hemodialysis treatment initiation and termination (Topic 7) appears as the largest node, indicating that procedural aspects of hemodialysis constitute a central theme in nursing documentation. Topics associated with clinical assessment and monitoring (Topics 2, 3, and 8) are closely linked to keywords such as vital signs, hypotension, bleeding, and vascular access, highlighting the importance of continuous patient monitoring and early detection of adverse events during hemodialysis.

Topics within the safety and risk management domain (Topics 1, 4, 5, and 6) are connected to keywords related to falls, bed environment, mobility, caution, nursing implementation, and safety education, including fire safety and evacuation preparedness. Notably, bed environment configuration and active nursing interventions for fall prevention appear as distinct yet closely related topics, suggesting that safety management in hemodialysis nursing encompasses both environmental control and direct nursing actions.

Overall, the network structure demonstrates that hemodialysis nursing records reflect an interconnected set of practices integrating treatment processes, clinical monitoring, and multilayered safety management.

### 3.4. Mapping of the 16‐Item Korean Nursing Surveillance Scale to Hemodialysis Nursing Records

Table 4 presents the mapping results of the 16‐item Korean Nursing Surveillance Scale based on 300,321 hemodialysis nursing records. From these records, only sentences reflecting surveillance‐related content were identified and included in the analysis. A total of 163,315 nursing record statements were identified, of which 147,341 statements (90.2%) were mapped to one or more surveillance items. The number of mapped nursing record statements differed across the 16 surveillance items. For each item, the table reports the total frequency of mapped statements, the frequency per item, and the percentage within each item. The within‐item mapping rates ranged from low values for some items to values exceeding 90% for others. Among the surveillance items, Item 7 (vascular access assessment) showed the highest total frequency, with 39,521 mapped nursing record statements and a within‐item mapping rate of 98.2%. For each surveillance item, representative nursing record statements corresponding to the mapped content are listed in the table.

**TABLE 4:** Mapping of the 16‐item Korean Nursing Surveillance Scale to hemodialysis nursing records.

Item number	Rank within item	Representative nursing record statements	Total frequency (*n*)	Frequency per item (*n*)	% (within item)
1	1	Hypotension and dizziness improved after intervention.	840	250	29.8
	2	Postdialysis, explained possible hypotension‐related dizziness and fall risk, and instructed the patient to remain on bed rest.		80	9.5

2	1	After discussing the patient’s condition with the nephrologist, decided to adjust UF to match dry weight.	5856	2507	42.8
	2	Discussed patient status with the nephrologist and decided to proceed with dialysis at net 0 kg for 4 h with HPR 500/300 IU.		2269	38.7

3	1	BP checked at 95/54 mmHg; placed patient in shock position, reduced BFR to 200 mL/min, and closely monitored BP.	1208	483	40.1
	2	Patient noted to be sweating; checked BST (blood glucose).		381	31.5
	3	VP suddenly increased to 300.		169	14.0

4	1	Explained that hypotension and dizziness may occur after dialysis, instructed the patient to rise slowly, and provided fall‐prevention education.	24,523	13,997	57.1
	2	Explained fall risk and provided safety care by locking bed wheels, raising side rails, and placing a fall‐precaution sign.		10,194	41.6
	3	Due to elevated VP and possible clot formation in the circuit, a set change was performed.		269	1.1

5	1	Under the HPR‐free protocol, infused NS 100 mL into the dialysis line every 20 min.	7272	7115	97.8
	2	Planned to check BP every 30 min and observe patient status.		29	0.4

6	1	Notified the nephrologist of the patient’s condition.	3125	3102	99.3

7	1	The vascular access site shows no erythema, pain, discharge, or warmth.	39,521	38,818	98.2
	2	No URI symptoms noted.		107	0.3

8	1	Patient reports having good bowel movements and that body weight has not increased much.	100	52	52.0
	2	Patient reports urine output decreased to about half of usual; lower‐extremity edema observed.		31	31.0

9	1	Patient reports falling at home this morning; slight bruising observed on the right palm.	186	96	51.6

10	1	The machine alarm sounded; checked the dialysis machine for abnormalities.	6970	4840	69.4
	2	Confirmed via SpO_2_ monitor that oxygen saturation remained stable at 100%.		1478	21.2
	3	Applied ECG monitoring and checked lead contact and waveform.		410	5.9

11	1	Checked vascular puncture site for signs of bleeding (oozing, hematoma, etc.).	19,373	14,363	74.1
	2	Vascular site shows no oozing, hematoma, or other signs of bleeding.		1159	6.0

12	1	Because of hypotension and dizziness, the patient is under close observation.	15,495	14,047	90.7
	2	On room air; patient denies dyspnea.		233	1.5

13	1	Checked patient status and gave handover to ward staff.	158	135	85.4

14	1	Vital signs checked.	32,535	25,970	79.8

15	1	Provided education on hemodialysis‐related self‐care (vascular access care, diet and fluid restriction, medication adherence, and coping with abnormal symptoms) and assessed the patient’s understanding and adherence.	3428	1576	46.0
	2	Explained the importance of independently checking vascular access status after dialysis.		439	12.8
	3	Today’s weight increased by 5 kg; explained the need for dietary and fluid self‐management.		401	11.7

16	1	Patient requested UF to be set to reach dry weight.	2725	1747	64.1
	2	Patient requested an additional 200 g UF because it is the weekend.		595	21.8

Total			163,315	147,341	90.2

## 4. Discussion

This study examined hemodialysis nursing records to clarify how nurses’ surveillance activities are patterned across the different phases of dialysis treatment. The keyword analysis identified terms such as “dialysis,” “condition,” “fall,” “check,” “pain,” “termination,” “initiation,” “vascular access,” “education,” and “prevention” as highly frequent, indicating that nursing documentation spans from predialysis assessment through intradialytic monitoring to postdialysis completion [27, 28]. This finding aligns with Wang et al. [27], who bibliometrically analyzed Web of Science data (2002–2023) and highlighted “vascular access complications,” “nurse‐led interventions,” and “self‐care behavior” as core clusters in HD nursing research, with emerging trends in symptom monitoring (e.g., pain and anxiety) [27]. Physiological surveillance was reflected by keywords including “dialysis,” “vascular access,” “blood pressure,” “vital signs,” and “bleeding” [29]. In contrast, terms such as “fall,” “pain”, “dizziness” [27], and “hypotension” pointed to a strong emphasis on patient safety–oriented observation care [30]. Previous reports from hemodialysis nurses likewise describe ongoing monitoring for hypotension, bleeding, and vascular access problems as a core aspect of safe care [31]. The frequent appearance of words such as “education,” “guidance,” and “explanation” suggests that communication and educational interventions are embedded in routine practice [27], which is consistent with research underscoring nurses’ role in promoting self‐care and treatment adherence among hemodialysis patients [29, 32]. The frequent appearance of the 30 most common keywords, accounting for 75.3% of all terms, underscores a structured and repetitive documentation pattern in hemodialysis nursing records [33]. For example, representative statements such as “The vascular access site shows no erythema, pain, discharge, or warmth” (Table 4) illustrate that nursing documentation is often structured in a checklist‐like format and frequently framed in negative (rule‐out) terms. This pattern suggests that nursing documentation tends to prioritize the confirmation of the absence of clinical problems rather than narrative description. This mirrors findings from recent studies on EHRs in renal care, where standardized checklists and embedded prompts facilitate consistent tracking of hemodynamic stability, vascular access integrity, and safety metrics [34].

Topic modeling of hemodialysis nursing records revealed three interconnected themes—“hemodialysis treatment process” (Topic 7), “clinical assessment and monitoring” (Topics 2, 3, and 8), and “safety and risk management” (Topics 1, 4, 5, and 6)—forming a cohesive rather than isolated structure [27]. Comparable interconnected topic distributions have emerged in topic modeling and network analyses of patient safety incident reports, where process‐oriented, monitoring‐focused, and safety‐centric clusters exhibit strong linkages [33]. The keyword–topic network in Figure 1 illustrates that documentation is organized around a treatment‐centered trajectory, with surveillance and safety activities embedded around this core rather than documented as separate tasks [27], supporting the conceptualization of NS as an integrated safety practice [33, 35]. This pattern aligns with prior investigations employing topic modeling on clinical free text to uncover latent co‐occurrence structures linking patient status and risk factors [36, 37]. Furthermore, the observation of keywords spanning multiple topics indicates semantic overlap, mirroring findings from text mining applications to safety narratives and EHR notes, where single terms contribute to diverse topics across clinical contexts [38, 39]. Given the inherent characteristics of free‐text nursing documentation, prior studies recommend incorporating supplementary natural language processing techniques—such as sentence‐level keyword co‐occurrence analysis or finer‐grained unit parsing—to improve the interpretability and robustness of topic modeling outcomes [40–44]. This pattern aligns with previous research inferring latent structures of co‐occurring concepts related to patient status and risk from clinical text [9, 41]. Moreover, keywords spanning multiple topics signal semantic overlap, consistent with text mining of free‐text safety narratives where identical terms contribute to diverse topics across clinical contexts [40, 45].

NS analysis revealed high documentation rates for vascular access site observations (NS7, 98.2%), vital sign measurement (NS14, 79.8%), bleeding checks (NS11, 74.1%), and hypotension/dizziness monitoring (NS12, 90.7%), underscoring nurses’ consistent emphasis on hemodynamic stability and access integrity during hemodialysis [27, 46]. These surveillance items align closely with the high‐frequency keywords and topics previously identified, including “vascular access,” “blood pressure,” “bleeding,” and “dizziness,” reflecting nurses’ pivotal role in detecting intradialytic hypotension and access‐related complications during hemodialysis sessions [27, 29, 46, 47]. For example, the vascular access sites represent a vital “lifeline” necessitating vigilant continuous monitoring, whereas vital signs serve as indispensable indicators for identifying adverse events like hypotension amid ultrafiltration processes [46–48]. Bleeding surveillance proves essential amid anticoagulant therapy in hemodialysis, with dizziness and hypotension emerging as prominent adverse effects, especially in older adults and those with diabetes mellitus [46, 49, 50]. In contrast, items related to patient self‐management and education, such as self‐care guidance (NS15), fall‐prevention education (NS4), and monitoring of body weight or urinary output (NS8), were documented less frequently [27, 51, 52]. Although “fall” ranked among the high‐frequency keywords in the initial analysis, fall‐prevention education appeared infrequently in the NS analysis, underscoring methodological disparities between keyword frequency metrics and structured surveillance item coding. This finding reflects a relative imbalance in the documentation of different types of nursing activities, rather than definitive evidence that such care was not provided. The observed documentation imbalance reflects differences in how nursing activities are recorded within EMR systems, rather than indicating differences in the care provided. This imbalance can be understood in terms of variations in the visibility of nursing activities within clinical documentation [53–55]. In hemodialysis settings, physiological surveillance activities are typically embedded within structured documentation fields that require routine entries, enhancing their consistency and visibility. In contrast, educational or supportive interventions are more often recorded in narrative formats, which are less standardized and may be less consistently represented in the documented record [54, 55]. This may suggest that documentation practices are associated with how different aspects of nursing work are represented within clinical records. Additionally, while factors such as clinical workflow demands or system‐level characteristics may be relevant [56], these interpretations remain inferential, as such factors were not directly examined in the present study and should therefore be interpreted with caution. Taken together, these findings should be interpreted as reflecting patterns in documentation practices, which may not fully capture the scope of care delivered. From a nursing management perspective, these results are meaningful because they indicate areas in which nurse leaders can improve documentation quality, standardize recording practices, and better align EMR design with both safety monitoring and patient‐centered care. Importantly, this study should be understood as an analysis of documentation practices, providing insight into how different aspects of nursing care are represented within clinical records rather than a comprehensive assessment of all care provided.

The integrated findings from keyword analysis, topic modeling, and surveillance item frequency provide a comprehensive analytic perspective on nurses’ surveillance practices, priorities, and contextualization within hemodialysis care [27, 29, 52]. These findings have implications for nursing leaders, including the development of surveillance‐oriented templates, structured EMR enhancements, and the use of documentation data for quality monitoring and decision‐making [29, 54].

This study has several limitations. First, the manual sentence‐level classification process relied on researchers’ clinical judgment, potentially introducing subjectivity in interpretation. Second, the analysis was confined to nursing records from a single medical institution, where unique organizational factors—such as the EMR system structure, documentation practices, and nursing workflow—may have influenced the results. Therefore, the findings may not be directly generalizable to other clinical settings with different documentation systems, institutional protocols, or nursing cultures. Third, only free‐text narrative nursing records authored by nurses were included, excluding surveillance activities captured in structured formats like checklists or flow sheets. Accordingly, this study reflects documented nursing activities rather than the full scope of care performed in practice, and certain interventions—particularly educational or supportive activities—may have been underrepresented in the records. Fourth, the use of LDA as a text‐mining approach presents inherent methodological limitations. Specifically, LDA does not account for contextual nuances such as negation or semantic polarity (e.g., distinguishing between “no bleeding observed” and “bleeding observed”), which may introduce ambiguity in topic interpretation. Although this limitation is less likely to affect the structured NS analysis, it remains a relevant constraint when interpreting the results of topic modeling.

To address these limitations and enhance the practical applicability of this study, the following recommendations are proposed. First, multi‐institutional data should be utilized to compare documentation patterns and surveillance activity recording across organizations, thereby assessing the generalizability and scalability of the identified patterns. Second, subsequent research is warranted to examine correlations between specific surveillance activities in nursing records—such as frequency and content—and clinical patient outcomes like fall incidence, vascular access complications, and treatment adherence, to evaluate the clinical validity of documentation practices. Third, to transcend keyword‐level analysis and achieve precise contextual interpretation, advanced natural language processing techniques based on sentence embeddings (e.g., BERT and sentence‐level contextual embeddings) should be incorporated for automated analysis of surveillance activity expressions. Fourth, collaboration between EMR system developers and frontline nurses is essential to design and implement surveillance‐centered structured templates that systematically capture repetitive and individualized nursing interventions, such as self‐management and fall prevention education. This approach would simultaneously alleviate documentation burden while ensuring consistent recording of core surveillance activities, thereby establishing a foundation for nursing quality management.

## 5. Conclusion

This study demonstrated that hemodialysis nursing documentation predominantly focuses on physiological surveillance and safety monitoring, as evidenced by high‐frequency keywords and topic clusters centered around vascular access, hemodynamic stability, and complication prevention. The integrated analysis revealed structured and repetitive documentation patterns. These patterns were aligned with documentation structures and safety priorities within EMR systems. However, documentation related to patient education and self‐management support appeared infrequent, indicating a relative imbalance in documentation emphasis rather than definitive evidence of differences in care delivery. These findings highlight the need to refine EMR systems through the development of more structured and actionable documentation templates. Specifically, in addition to fields for vital signs, vascular access assessment, and complication monitoring, EMR templates should incorporate clearly defined prompts or checkboxes for the rapid and standardized documentation of patient education and self‐management support, such as predefined categories for fall prevention, fluid restriction, medication adherence, and symptom reporting. Such template enhancements may facilitate more balanced documentation of both physiological surveillance and patient‐centered educational interventions, while also reducing variation in recording practices. From a nursing management perspective, these findings provide actionable insights for nurse leaders to improve documentation quality, standardize recording practices, and support data‐driven decision‐making in clinical and organizational management. Future studies should leverage multi‐institutional data to explore documentation trends, correlate surveillance records with patient outcomes, and deploy sentence‐level NLP to capture nursing judgment semantics. Importantly, these findings should be interpreted as reflecting documentation practices, which may not fully capture the scope of care delivered.

## Author Contributions

Mi‐Kyoung Cho: conceptualization; formal analysis; methodology; supervision; writing–original draft; and writing–review and editing. Hye‐Young Kim: conceptualization; resources; and data curation. Hyohjung Lee: investigation and data curation. Yoon Hee Cho: conceptualization; formal analysis; methodology; visualization; writing–original draft; and writing–review and editing.

## Funding

This research received no external funding.

## Disclosure

All authors reviewed and approved the final version of the manuscript.

## Conflicts of Interest

The authors declare no conflicts of interest.

## Supporting Information

Additional supporting information can be found online in the Supporting Information section.

## Supporting information


**Supporting Information** Supporting Information 1. STROBE checklist for cross‐sectional studies used to guide the reporting of this study.

## Data Availability

The data that support the findings of this study are not publicly available due to institutional and ethical restrictions but are available from the corresponding author upon reasonable request and with permission of the data‐providing institution.
